# Bidirectional interplay between gut and liver in inflammatory bowel disease and hepatobiliary conditions

**DOI:** 10.1007/s10238-026-02102-w

**Published:** 2026-04-07

**Authors:** Ali Abdulla, Ayan M. Sheikhnoor, Kevin Ferrao, Lucy I. Stiles, Kosha J. Mehta

**Affiliations:** 1 Faculty of Life Sciences and Medicine, GKT School of Medical Education, King’s College London, London, UK; 2 Faculty of Life Sciences and Medicine, Centre for Education, King’s College London, London, UK

**Keywords:** Inflammatory bowel disease (IBD), Liver disease, Gut-liver axis, Metabolic dysfunction-associated steatotic liver disease (MASLD), Alcohol-related liver disease (ARLD), Primary sclerosing cholangitis (PSC), Comorbidities

## Abstract

Inflammatory bowel disease (IBD) can affect extraintestinal organs, including the liver. The bidirectional relationship between the gut and liver underpins the interplay between IBD and liver pathologies; both are predicted to rise. This review discusses the anatomical-physiological context of the gut-liver axis, the influence of IBD on the liver and vice versa, and hepatobiliary conditions co-existing with IBD, namely, MASLD, ARLD, PSC and gallstones. About 70% of the liver’s blood supply comes from the gut via the portal vein, which carries both nutrients and gut-derived microbial products, as regulated by the intestinal barrier. IBD is associated with gut dysbiosis and compromised intestinal barrier integrity (both exacerbated by alcohol consumption, an IBD risk factor), allowing microbial translocation to the liver, which triggers hepatic inflammation/injury. This exacerbates pre-existing liver disease or increases its risk; for example, IBD increases MASLD risk. However, translocated lipopolysaccharides may alleviate cholestatic liver injury, enabling IBD to ameliorate PSC. In turn, PSC can promote a favourable gut microbiota profile to alleviate IBD. The liver maintains gut homeostasis/microbiota through bile acid secretion. Disrupted bile acid secretion in gallstones increases IBD risk, while disrupted bile acid reabsorption in IBD increases gallstone risk. Disrupted bile acid metabolism and an abnormal gut microbiota in MASLD may exacerbate the IBD course. For co-existing IBD-hepatobiliary pathology, management strategies are unestablished, and adverse effects of IBD therapeutics on hepatobiliary pathologies are observed (and vice versa). This review facilitates a structured understanding of the pathophysiological connections and may inform/improve the current management of coexisting IBD-hepatobiliary conditions.

## Introduction

Inflammatory bowel disease (IBD) is a collection of remitting-relapsing or progressive inflammatory conditions of the gastrointestinal tract [1]. Crohn’s disease and ulcerative colitis are the two most recognised disorders of IBD. A dynamic interaction between genetic susceptibility, immunological imbalance, and microbial shifts in the gut influences the clinical occurrence of Crohn’s disease and ulcerative colitis [2], in addition to environmental triggers such as smoking, diet, and stress [3].

Clinically, both Crohn’s disease and ulcerative colitis typically present with abdominal pain, weight loss and fever. Ulcerative colitis more characteristically manifests with bloody diarrhoea with or without mucus [4–6]. These conditions can be distinguished by their histology, patterns of inflammation and anatomical distribution. For example, non-caseating granulomas detected in a biopsy are highly suggestive of Crohn’s disease [6]. On the other hand, crypt abscesses, loss of goblet cells, and distortion of crypt architecture are noticed in ulcerative colitis [4, 5]. In Crohn’s disease, it has been established that there are intermittent patches of transmural inflammation throughout the gastrointestinal tract. These can occur anywhere from the mouth to the anal region, although the distal ileum is most commonly affected [6, 7]. Contrastingly, in ulcerative colitis, inflammation is continuous, and it has been traditionally conceptualised as localising to the mucosal layer of the colon and the rectum [8].

However, emerging evidence challenges this longstanding dogma by recognising transmural changes within the ulcerative colitis population. Intestinal ultrasound studies have enabled the exploration of key characteristics (loss of haustration, thickened bowel wall, loss of bowel wall stratification, fibrofatty mesenteric changes and submucosal thickening) that support deeper ulcerative colitis disease activity [9, 10]. Such transmural insights are further corroborated by macroscopic and histological observations like colonic shortening, excess fat deposition, fibrotic shifts and strictures, muscular remodelling, muscularis mucosae thickening and submucosal fibrosis. These transmural changes can justify some of the observed progressive structural damage and functional symptoms seen in ulcerative colitis patients. For example, the transmural alterations may induce diarrhoea, urgency, incontinence, and impact the neuromuscular dimension of the bowels, allowing for altered bowel motility in these patients [9]. Broadly, the transmural features of ulcerative colitis are not as extensive as the features of complicated Crohn’s disease (intestinal fistulas, intra-abdominal abscesses, or inflammatory masses) [7, 9]. Nevertheless, re-evaluation of the traditional mucosal-centric paradigm in ulcerative colitis can guide future treatment initiatives. Inspiration for the development of transmural-monitoring approaches for ulcerative colitis can be sought from the STRIDE-II project, which prompts a discussion on transmural healing as a potential marker for Crohn’s disease remission [10].

In IBD, while gastrointestinal symptoms remain central to disease presentation, it is widely appreciated as a systemic illness, highlighted by the occurrence of extraintestinal manifestations in up to 50% of patients throughout their disease course [11]. Multiple organ systems could be affected, including musculoskeletal (peripheral and axial inflammatory arthritis), dermatological (erythema nodosum, pyoderma gangrenosum), and ocular (uveitis, episcleritis) [12].

## Rationale for this topic: the underlying connections

The gut and the liver share an intricate bidirectional relationship, both under physiological and pathological conditions. The dysfunction of one can directly affect the other [13].

### The bidirectional connection

Under physiological conditions, the liver actively shapes intestinal immunity and microbiota composition through enterohepatic circulation, including mechanisms mediated via bile acids [14]. Accordingly, in a liver disease, if there are perturbations in bile acid excretion, then the gut microbiota can be altered, leading to gut dysbiosis [15–17]. Furthermore, decreased bile acid excretion can disrupt gut functions such as the digestion and absorption of fats, as well as compromise the intestinal barrier [18].

In the other direction, under physiological conditions, the gut provides nutrient-rich blood to the liver, serves as a barrier between the gut microbiota and the liver, and prevents inappropriate translocation of microbial products via the portal vein, thereby averting subsequent liver inflammation. Also, hormones secreted in the gut affect bile acid regulation and glucose metabolism [19–22]. However, under intestinal pathological states, for example, in IBD, chronic intestinal inflammation exposes the liver to microbial and immune-derived signals, contributing to hepatic complications [13, 23]. Unsurprisingly, there is a significant liver involvement in IBD, where it is an important extraintestinal manifestation of IBD, with 30% of IBD patients showing abnormal liver tests and 5% progressing to the development of chronic liver disease [24].

### Angle of co-existing prevalence

Amongst the hepatobiliary manifestations in IBD, metabolic dysfunction-associated steatotic liver disease (MASLD) is the most common, affecting around 26.1% of IBD patients, followed by 4.1% with biliary stones and 1.67% with primary sclerosing cholangitis (PSC) [24, 25]. This emphasises the varied and significant burden of liver involvement in the IBD population. So far, the link between PSC and IBD is the most well-studied [26] and is considered the prototypical gut-liver axis disease [14]. However, less is known about other liver issues that can coexist with IBD, such as MASLD [26], the most common chronic liver disease globally [27].

### Therapeutic perspectives

Despite these evident connections, tailored management for co-existing IBD-liver pathology or IBD-biliary pathology remains to be established; there is no confirmed national consensus around this. In most instances, the co-existing conditions are treated simultaneously but separately, which can cause adverse effects in some patients with co-existing IBD-hepatobiliary pathology [28, 29]. Moreover, certain IBD medications, including immunosuppressive and biologic therapies, can cause drug-induced liver injury and add to the complexity of managing the liver condition in IBD cases [30]. Unsurprisingly, the gut–liver axis has increasingly emerged as a potential framework for understanding how intestinal inflammation in IBD may propagate liver dysfunction and vice versa.

### The essence

Considering the aforementioned links, alongside data that IBD and liver conditions, such as alcohol-related liver disease (ARLD), are expected to increase worldwide [27, 31], it is essential to examine findings on the various hepatobiliary issues in the context of IBD. Therefore, this review examines the relationship between the gut and the liver in a healthy state and discusses the pathological association between IBD and hepatobiliary conditions. This will not only allow a systematic understanding of the context but can also guide the current management of coexisting IBD-hepatobiliary pathologies, thereby improving prognosis in these patients.

The hepatobiliary conditions reviewed here are the most common conditions co-existing with IBD, namely, MASLD, ARLD, gallstones and PSC.

## Connections between the gut and liver

### Anatomical connection between the gut and the liver

The liver is anatomically and functionally positioned to receive continuous input from the gastrointestinal tract, forming the foundation of the gut–liver axis [32].

Approximately 70% to 75% of the liver’s blood supply is delivered via the portal vein, which drains the gastrointestinal tract via the celiac, superior mesenteric, and inferior mesenteric veins [19]. In parallel to the portal system, there is an indirect connection between the gut and the liver via the mesenteric lymphatic system. This begins in the small intestinal lacteals (capillaries in the intestinal villi) that collect chyle, a lymph rich in chylomicrons [33] and fat-soluble vitamins. This fluid ultimately drains into the cisterna chyli at the base of the thoracic duct [34]. From here, it empties into the junction of the left subclavian and internal jugular veins and ultimately connects to the systemic venous circulation [35]. This venous blood then enters the heart, and following oxygenation, it reaches the liver via the hepatic artery, which accounts for 25–30% of the liver’s blood flow [36, 37]. This mesenteric lymphatic network transports absorbed lipids and solutes, such as L-carnitine, to the liver, offering an additional route through which the intestine modulates liver physiology [37].

### Gut-liver axis: immunological connections through the layers of the intestinal barrier

Forming a boundary between the gut microbiota and the liver, the intestinal barrier is structurally composed of three distinct layers.

The layer closest to the intestinal lumen is the mucus barrier. This barrier is particularly thick in the colon, where microbial density is relatively high. It provides a niche and nutrition for bacteria, while for the host, it functions as the first line of defence. Accordingly, most of the interactions between the host and the gut microbes are indirectly mediated by the microbial products and metabolites released by the gut microbiome. In the colon, the mucus barrier consists of two sublayers: an external mucus layer, which is adjacent to the intestinal lumen and an internal mucus layer, which is next to the intestinal epithelium. Bacteria can attach to the external mucus layer, allowing them to survive in the gut despite peristaltic movements. In contrast, the internal mucus layer is nearly sterile due to the presence of antimicrobial peptides and size-limiting meshes, which prevent bacterial penetration into the inner mucus layer [20].

The middle layer of the intestinal barrier is the epithelial layer. It is composed of a single layer of intestinal epithelial cells, including goblet cells, enterocytes, enterochromaffin cells, and tuft cells. This layer serves as a physical, electrical, and chemical barrier. The physical property of the barrier arises from tight junctions on the lateral side of the intestinal epithelial cells, which secure these cells together and prevent the passage of materials between the cells. The electrical barrier arises from the negative charge of the intestinal brush border, and the chemical barrier is due to the release of antimicrobial peptides by the intestinal epithelial cells [20].

The innermost layer of the intestinal barrier is the gut vascular barrier. If the epithelial barrier is breached, then the gut vascular barrier inhibits bacterial entry into the portal circulation, preventing subsequent dissemination in the systemic circulation. In addition to the structural characteristics of the intestinal barrier discussed so far, the barrier also consists of immune cells, including intraepithelial and lamina propria immune cells, and soluble mediators, including immunoglobulin A (IgA) and antimicrobial peptides. Essentially, all these components contribute to the boundary between the gut microbiota and the liver [20].

### Gut-liver axis: further immunological connections

Given the constant exposure of the intestinal epithelium to microbial antigens and its direct drainage into the portal circulation, immune surveillance at this interface is critical for preventing inappropriate immune activation in the liver [38]. Some cells have emerged as key immunological players in this process, for example, the Tissue-resident memory T cells (Trm-T cells). Unlike central and effector memory T cells, Trm-T cells are memory T cells that persist in peripheral tissues without moving into the systemic circulation. These cells express tissue residence markers such as CD69 and CD103; the latter pairs with β7 to form αEβ7 heterodimers that bind to E-cadherin on intestinal epithelial cells, thereby facilitating Trm-T-cell retention within the epithelium. In a healthy intestine, Trm-T cells contribute to defence against pathogens and show tolerance to dietary antigens. These cells can reinforce epithelial barrier integrity and prevent bacterial translocation by inducing protective inflammation. Upon reinfection, these cells rapidly eliminate the pathogens via proliferation, cytokine secretion, and recruitment of additional immune effectors. Trm-T-cell-derived cytokines, such as TNF-α and IL-2, activate the surrounding immune cells to protect the host [39].

Additionally, innate lymphoid cells (ILCs) play a crucial role in intestinal immune homeostasis. Specific ILC sub-populations display distinct regional distributions throughout the gastrointestinal tract. For instance, the ILC1s are predominantly found in the upper gastrointestinal regions, such as the oesophagus, stomach, and duodenum, whereas NKp44^+^ ILC3s predominate in the lower gastrointestinal tract, including the ileum and colon [40].

In the small intestine, Paneth cells in the epithelial crypts produce antimicrobial peptides, including alpha-defensins and regenerating islet-derived 3-gamma, contributing to innate intestinal defence [40].

In addition to innate immunity, adaptive immune cell populations also demonstrate marked regional specialisation. For example, compared to the ileum, the healthy colon exhibits higher levels of regulatory T cells (Tregs), T helper-17 (TH17) cells, and double-negative T cells. Also, compared to the ileum and left colon, the right colon has a slightly higher percentage of Treg cells in healthy mucosa. When compared to the ileal tissue, the colon displays a regional enrichment of TH17 cells, while the proportion of TH1 cells is uniformly distributed across the intestinal compartments [41].

The functionality of all these cell types in the intestinal region further contributes to the boundary between the gut microbiota and the liver.

### Gut-to-liver: maintenance of a tolerogenic state in the liver

**Fig. 1:**
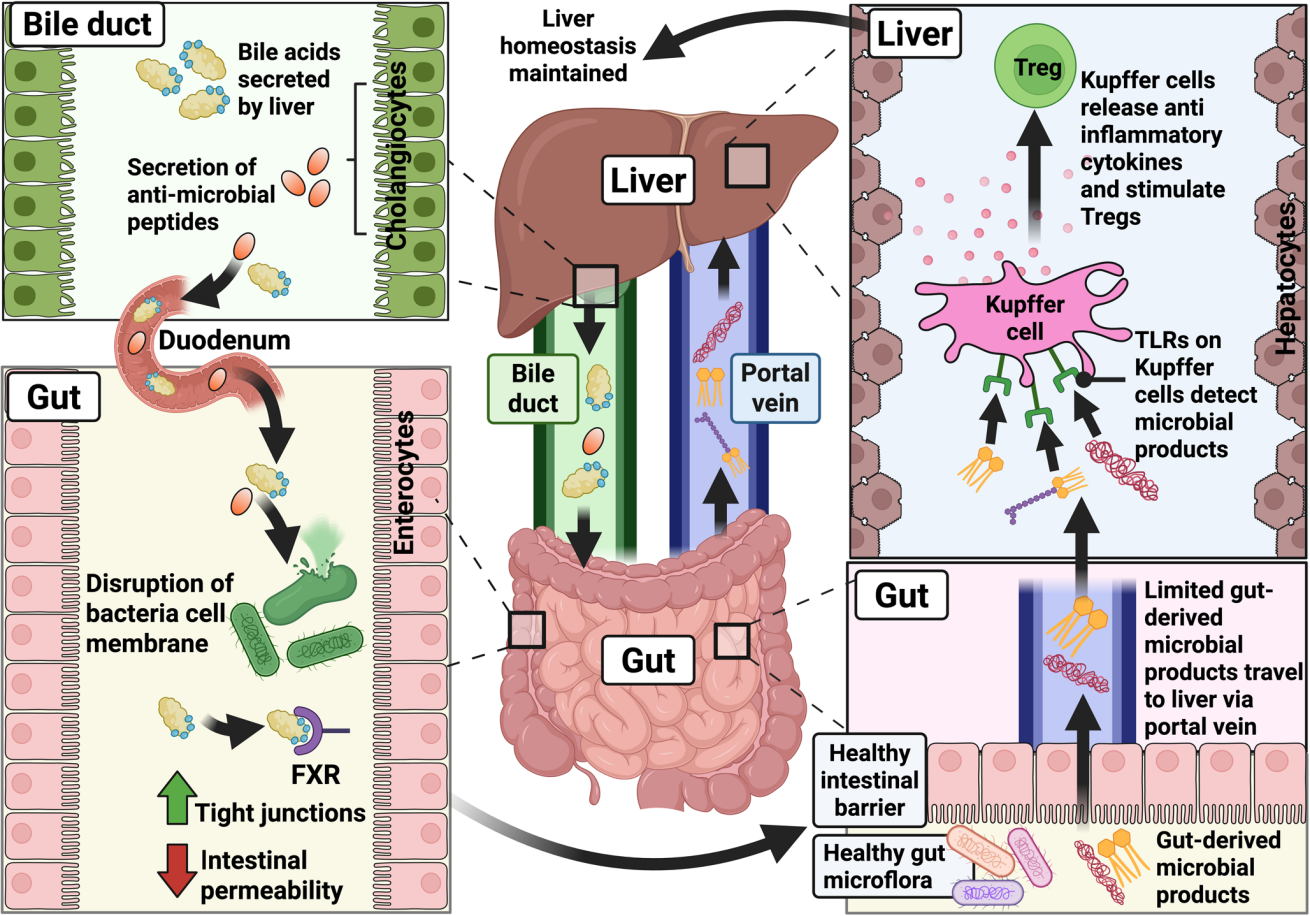
**Physiological connections between the gut and liver: Immunological links** A healthy intestinal barrier and gut microflora enable controlled translocation of microbial products such as short-chain fatty acids, bacterial DNA, and lipopolysaccharides from the gut to the liver via the portal vein. In the liver, Kupffer cells detect these products through TLRs but do not trigger inflammation. Instead, they produce an anti-inflammatory response by stimulating Tregs and releasing IL-10. Overall, this helps maintain a tolerogenic state. A healthy liver produces bile acids, and cholangiocytes in the bile ducts secrete antimicrobial peptides. These bile acids and peptides are released into the duodenum, where they disrupt bacterial membranes and cause bacterial cell death, thereby modulating the gut microflora. Additionally, in the gut, bile acids activate the farnesoid X receptor (FXR), which upregulates tight junctions and consequently decreases intestinal permeability, contributing to a healthy intestinal barrier. The green upward arrow in the figure indicates an increase, whereas the red downward arrow indicates a decrease. This figure was created with BioRender.com.

Due to the blood flow from the gut to the liver, the liver is exposed to a variety of gut-derived microbial materials (Fig. 1). Therefore, it is important to prevent inappropriate and/or overstimulation of the immune response in the liver to maintain physiological homeostasis. The gut-derived bacterial products include pathogen-associated molecular patterns (PAMPs), such as lipopolysaccharides, lipoteichoic acid, and components from bacterial DNA. These also include microbe-associated molecular patterns (MAMPs), such as short-chain fatty acids (SCFAs), as well as danger-associated molecular patterns (DAMPs) [14]. SCFAs and some other microbial metabolites have an immunomodulatory potential that can enhance intestinal barrier function, which may encourage liver repair [42]. On the other hand, products such as lipopolysaccharides have the potential to activate innate immune responses despite being derived from commensal microbiota [43]. To avoid inappropriate immune activation in the liver, Kupffer cells, the resident macrophages located in the hepatic sinusoids, help maintain a state of tolerance. These cells express pattern recognition receptors, such as Toll-like receptors (TLRs), which detect gut-derived antigens while remaining non-inflammatory under physiological conditions. Kupffer cells promote immune homeostasis by producing anti-inflammatory cytokines, such as interleukin 10 (IL-10), and stimulate regulatory T cells (Tregs). This tolerogenic environment ensures that the liver can process microbial signals from the gut without triggering pathogenic immune responses [21, 44] (Fig. 1).

### Liver-to-gut: bile acid production helps lipid emulsification in the gut

Hepatocytes synthesise the primary bile acids, cholic acid and chenodeoxycholic acid from cholesterol [45]. These bile acids are subsequently conjugated with taurine or glycine and secreted into the bile canaliculi via the bile salt export pump (BSEP/ABCB11) to reduce their cytotoxicity in the liver. Conjugated bile acids are transported through the biliary tract and released into the duodenum, where they facilitate the emulsification and absorption of dietary lipids. In the intestinal lumen, primary bile acids are metabolised by the gut microbiota into more than 20 secondary and tertiary bile acid species, the most prominent ones being deoxycholic acid and lithocholic acid, produced via bacterial 7α-dehydroxylation of cholic acid and chenodeoxycholic acid, respectively [45–48]. Notably, the interaction between the gut microbiota and bile acids is bidirectional. In mouse models, gut bacteria can influence the bile acid pool through bile salt hydrolase, and bile acids can, in turn, influence the microbiota composition, as detailed in the subsequent section. [14].

Most bile acids are reabsorbed into the intestinal epithelial cells of the terminal ileum and returned to the liver via the portal circulation, thereby completing the enterohepatic circulation. Approximately 5% of bile acids escape reabsorption in the intestine and undergo modification into secondary bile acids by colonic bacteria [49, 50]. In this enterohepatic circulation, bile acids can be recycled up to 12 times per day among liver hepatocytes and intestinal enterocytes [51].

### Liver-to-gut: protective effect of bile acids and the role of activated FXR in the intestine

Cholangiocytes, the epithelial cells lining the bile ducts, contribute to gut–liver homeostasis by modifying bile composition and balancing its pH. This includes the secretion of antimicrobial peptides (e.g. β-defensin 2 and cathelicidin), IgA, and cytokines into the bile [52–54]. These actions enhance the immunological and biochemical properties of the bile secreted into the intestine, thereby supporting the normal intestinal barrier function (Fig. 1).

Bile released from the liver can influence the composition and behaviour of the gut microbiota (Fig. 1). For example, unconjugated bile acids show strong antibacterial activity, with gram-positive bacteria being more susceptible than gram-negative bacteria. Essentially, bile acids possess amphipathic and detergent properties that can disrupt bacterial cell membranes, cause DNA damage, and alter protein conformation, causing cytotoxic effects on the bacteria. Bile acids can also affect bacterial metabolism in the gut [55].

In addition, bile acids activate the nuclear farnesoid X receptor (FXR) present in the intestinal epithelial cells (Fig. 1), which causes the following outcomes: (a) FXR upregulates tight junctions in the intestinal epithelial cells, thereby decreasing intestinal permeability and contributing to maintaining a healthy intestinal barrier [20]. (b) FXR increases the production of anti-inflammatory cytokines, such as IL-10, and inhibits pro-inflammatory cytokines, such as IL-1, in the intestinal mucosa [56]. Furthermore, a pre-clinical study showed that an FXR agonist regulates genes involved in antimicrobial defence and immune modulation, such as the genes encoding angiogenin-1 (Ang1) and inducible nitric oxide synthase, in the ileum [57]. Thus, FXR helps regulate host immune responses.

### Gut-to-liver: intestinal FXR activation via FGF-19 mediates bile acid regulation and glucose metabolism in the liver

**Fig. 2:**
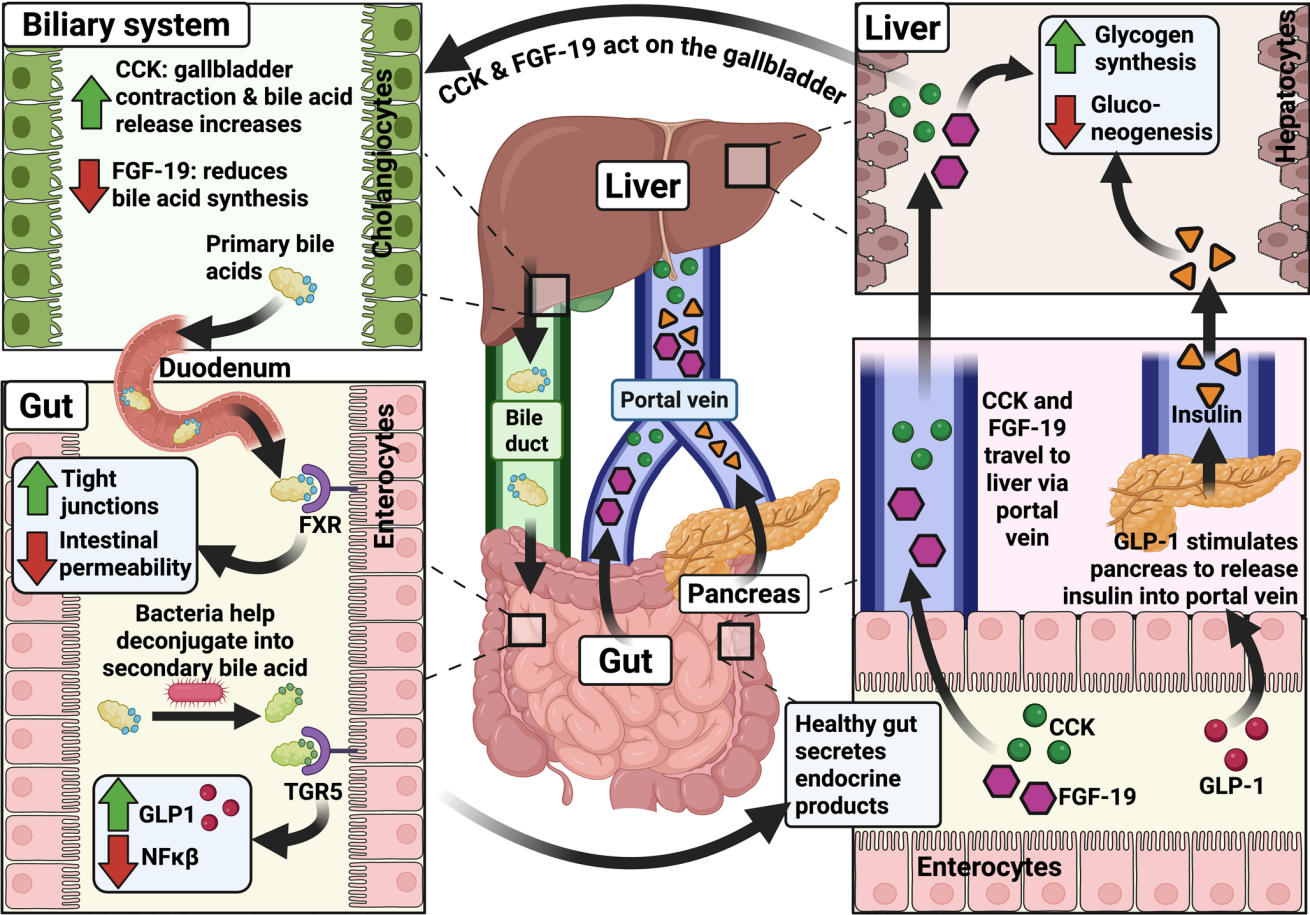
**Physiological connections between gut and liver: Endocrine and metabolic links** Following nutrient ingestion, GLP-1 secreted by intestinal L-cells enhances insulin secretion and inhibits glucagon release from the pancreas. Insulin travels to the liver via the hepatic portal vein and acts directly on hepatocytes to reduce gluconeogenesis and promote glycogen storage. FGF-19 and CCK travel from the gut to the liver via the hepatic portal vein. In the liver, FGF-19 suppresses CYP7A1, subsequently reducing bile acid synthesis, whilst CCK stimulates gallbladder contraction and promotes the release of bile acids into the intestine. In the intestine, bile acids activate FXR and promote epithelial integrity by upregulating tight junctions. The secondary bile acids (deconjugated from primary bile acids by bacteria) activate Takeda G-protein-coupled receptor 5 (TGR5), which increases GLP1 secretion and decreases the production of NFkβ. Collectively, these endocrine pathways maintain gut-liver metabolic homeostasis. The green upward arrow in the figure indicates an increment, whereas the red downward arrow indicates a decrement. This figure was created with BioRender.com.

Hormones secreted in the gut affect bile acid regulation and glucose metabolism in the liver. For example, following bile-acid–mediated activation of the FXR, the ileal enterocytes secrete the hormone Fibroblast growth factor-19 (FGF-19) [21, 58]. FGF-19 travels via the portal circulation [59] to the liver and binds to the βKlotho–FGFR4 receptor complex on the hepatocytes. This reduces the expression of cholesterol 7α-hydroxylase (CYP7A1) (the rate-limiting enzyme in bile acid synthesis) and cytochrome P450 12α-hydroxylase B1 (CYP8B1) in the liver, collectively suppressing hepatic bile acid synthesis. This negative feedback loop, which regulates bile acid synthesis, not only protects hepatocytes from bile salt-mediated toxicity but also enhances the liver’s capacity to manage bile acid overload. Alongside, FGF-19 acts independently of insulin to reduce hepatic gluconeogenesis and promote glycogen synthesis (Fig. 2) [14, 21, 22, 60, 61]. The binding of bile acids to TGR5 in the intestine also helps modulate glucose metabolism, energy expenditure, and inflammatory responses across multiple tissues, including the liver (Fig. 2) [62].

### Gut-to-liver: role of the intestine-secreted GLP-1 in hepatic glucose homeostasis- further endocrine and metabolic links between gut and liver

Endocrine signalling from the gut plays a central role in regulating hepatic function (Fig. 2). For example, following nutrient ingestion, glucagon-like peptide 1 (GLP-1) secreted by intestinal L-cells enhances glucose-stimulated insulin release from the pancreas and inhibits glucagon secretion [63, 64]. Insulin is transported to the liver via the hepatic portal vein [65]. This eventually increases glycogen synthesis and decreases gluconeogenesis in the liver (Fig. 2). Other gut-derived hormones contribute additional regulatory layers to the gut-liver axis. For example, Cholecystokinin (CCK) is released from intestinal I-cells in response to dietary fat. It induces gallbladder contraction and sphincter of Oddi relaxation, promoting bile release and subsequent lipid emulsification (Fig. 2). Peptide YY is co-secreted with GLP-1 by enteroendocrine L-cells in the distal ileum and colon. It modulates gastrointestinal motility and alters pancreatic insulin secretion, thereby affecting nutrient delivery and hepatic metabolic input [66–69].

## Effect of IBD on the liver, and liver disease on the gut

### Influence of IBD on a healthy liver

**Fig. 3:**
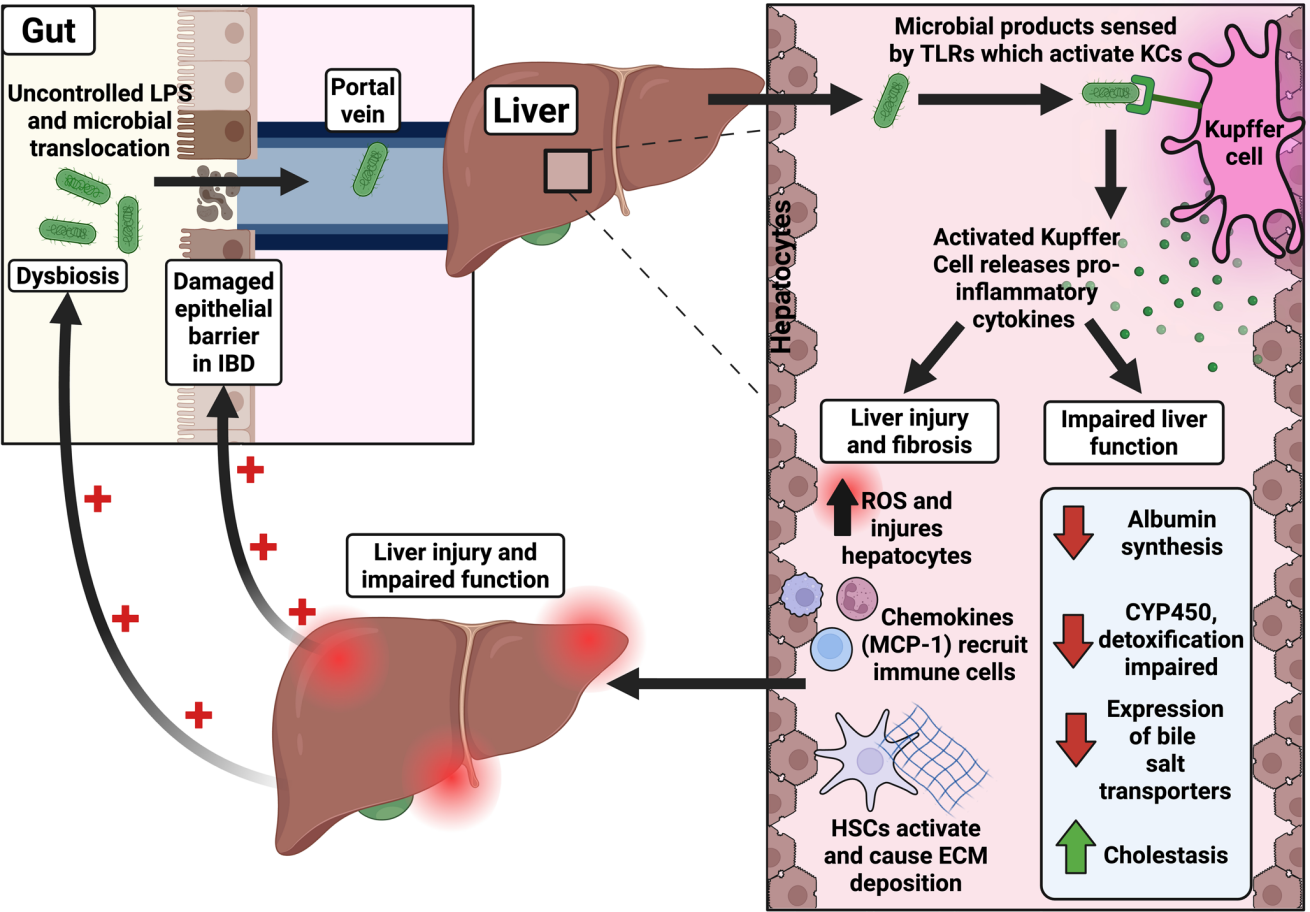
**The effect of IBD on liver function** In IBD, dysbiosis and epithelial barrier dysfunction increase intestinal permeability, allowing uncontrolled translocation of microbial products such as lipopolysaccharide and bacterial DNA into the portal vein. Within the liver, these products are recognised by TLRs on Kupffer cells, leading to their activation and subsequent release of pro-inflammatory cytokines. This increases reactive oxygen species, which injure hepatocytes, chemokines such as MCP-1 recruit immune cells and aggravate inflammation, and the activated hepatic stellate cells (HSCs) promote excessive extracellular matrix deposition leading to fibrosis. Concurrently, impaired hepatocyte function decreases albumin synthesis, reduces cytochrome P450 expression, and downregulates bile salt transporters, causing cholestasis. Hepatic inflammation and dysfunction can perpetuate gut permeability and dysbiosis, creating a self-perpetuating inflammatory cycle between the gut and liver. The green upward arrow in the figure indicates an increment, whereas the red downward arrow indicates a decrement. The red cross signs represent further exacerbation of the existing intestinal pathology. This figure was created with BioRender.com.

Since the liver receives approximately 70% of its blood supply from the portal vein [70], which drains the gastrointestinal tract [71], IBD is likely to have significant effects on liver function, particularly when there is chronic intestinal inflammation and dysbiosis in IBD (Fig. 3).

As detailed previously, a healthy intestinal barrier prevents the uncontrolled translocation of microbial products from the intestine into the portal vein, thereby preventing inappropriate immune activation in the liver (Fig. 1). In IBD patients, the integrity of the intestinal mucosal barrier is compromised [72] (Fig. 3), making the intestinal barrier hyperpermeable. This promotes excessive translocation of microbial products like lipopolysaccharides from the gut to the liver through the portal circulation [73]. In the liver, the Kupffer cells detect these microbial products. Lipopolysaccharides and lipoteichoic acid, components of gram-negative and gram-positive bacteria, respectively, can engage with TLRs on the Kupffer cells and stimulate innate immune responses. These cells subsequently release a range of pro-inflammatory cytokines (such as TNF-α, IL-1β, IL-6), triggering a cascade of inflammatory responses within the liver [74], thereby promoting liver fibrosis and cancer [51].

These Kupffer cell-derived pro-inflammatory cytokines induce oxidative stress through the generation of reactive oxygen species (ROS), damaging hepatocytes and impairing mitochondrial function [75]. These also inhibit the synthesis of hepatocyte albumin, a key liver-produced protein [76]. Furthermore, cytokines downregulate the expression of CYP450 [77], a key enzyme in liver detoxification [78], and also downregulate the expression of bile salt transporters, which contribute to cholestasis [79]. In addition to affecting hepatocyte function, chemokines such as MCP-1 recruit immune cells, namely monocytes, macrophages and T cells, which fuel these inflammatory responses [80] (Fig. 3).

In chronic liver injury, cytokines such as TGF-β and PDGF promote the transdifferentiation of hepatic stellate cells (HSCs) into myofibroblast-like cells. These activated HSCs generate an excessive amount of extracellular matrix, including collagen I, collagen III, laminin, and fibronectin [51]. This causes further hepatocellular injury, leading to liver fibrosis [81]. Activated Kupffer cells can also produce thromboxane A2, a vasoconstrictive eicosanoid that contributes to increased intrahepatic vascular resistance and the development of portal hypertension [82].

T cells in the intestine express α4β7 integrin and chemokine receptors such as CCR9 (showcasing a gut-homing profile). During their movement to lymph nodes for maturation, these receptors guide the T cells back to the intestine via interactions with MAdCAM-1 and CCL25 on the intestinal endothelium. In IBD with certain co-existing liver diseases, such as PSC, the hepatic endothelium aberrantly expresses MAdCAM-1 and gut-associated chemokines. This misdirects T cells from the gut into the liver, further contributing to hepatic inflammation [83].

Collectively, these mechanisms illustrate how intestinal inflammation in IBD can disrupt hepatic homeostasis through (a) the activation of Kupffer cells, ultimately impairing liver function even in the absence of a defined hepatobiliary disease and (b) the misdirection of the intestinal T cells to the liver (Fig. 3).

### Macrophages in chronic liver disease

Hepatic macrophage population comprises resident Kupffer cells and monocyte-derived macrophages. Under physiological conditions, Kupffer cells play an active role in the maintenance of liver homeostasis [84, 85]. Their activation underlines the relationship between microbial translocation and intrahepatic immune activation [74]. However, chronic liver disease has been associated with dynamic remodelling of the hepatic macrophage pool [84]. During severe injury, the embryonic Kupffer cell population is reduced and replenished by infiltrating monocyte-derived macrophages (MoMFs) [86], as seen in animal models of chronic liver injury, where MoMFs made up the bulk of liver macrophages [85]. Also, Kupffer cell reduction has been described in settings of hepatic insult secondary to pathogens (e.g., murine cytomegalovirus, vaccinia virus or bacterium *Listeria monocytogenes*), diet-induced non-alcoholic steatohepatitis and hepatocellular carcinoma animal models [84, 87]. Even in patients with alcohol-associated cirrhosis or severe alcohol-associated hepatitis, there was an approximate 90% reduction in Kupffer cells, as evidenced by downregulation of Kupffer cell signature markers (MARCO, CD5L, and TIMD4) and concurrent upregulation of MoMF-associated genes (TREM2, GPNMB, and SPP1) expression [88].

A central role of Kupffer cells is to conduct phagocytosis of invading pathogens and dead or apoptotic cells [89–91]. Relative to the homeostatic processes of Kupffer cells, MoMFs maintain a significantly compromised phagocytic capacity [89, 92]. Thus, MoMFs of the chronically injured liver may differ from Kupffer cells in their response to microbial antigens and may show diminished immunoregulatory functions compared to Kupffer cells. This shift in macrophage composition has been linked with long-term hepatic immune dysregulation that could promote susceptibility to bacterial infections, exacerbate ongoing tissue damage, and impair the recovery of hepatic function [86, 88, 92]. These findings invite comprehensive studies to explore the extent of MoMF prevalence throughout various aetiologies of hepatic pathology. Such studies could guide the development of therapeutic strategies and assess the possibility of defining prognostic markers that integrate macrophage populations.

### Influence of liver disease on a healthy gut

Liver disease can affect the microstructure and function of the gastrointestinal tract through several interconnected mechanisms.

Firstly, in liver diseases with reduced bile excretion, such as in cholestatic liver disease, there is a disruption in the production, flow, and recycling of bile acids, ultimately reducing the amount of bile acids reaching the intestine [93]. This results in impaired fat digestion and absorption from the gastrointestinal tract. A further consequence of reduced bile acid flow into the intestine is the decreased activation of FXR and TGR5, which normally play key roles in promoting mucosal immunity and maintaining the intestinal barrier [18] (Figs. 1 and 2). Reduced expression of tight junction proteins, such as zona occludens-1, seen in MASLD and ARLD, can also damage the intestinal epithelial barrier [94, 95].

Secondly, liver disease can induce changes in the composition of the gut microflora. This could be due to a combination of changes in bile acid secretion, chronic inflammation, and immune dysfunction, which promote the overgrowth of pathogenic microbes in the gut. In addition, poor nutrition and frequent use of antibiotics in patients with liver disease can contribute to gut dysbiosis [15–17]. In turn, this will exacerbate liver inflammation/injury due to gut-derived microbial products, thereby reinforcing the cyclical nature of the gut-liver axis mechanisms [96].

The following sections review the mechanistic and therapeutic angles of IBD in association with some of the most common hepatobiliary conditions.

## MASLD/NAFLD and IBD

### Overview of MASLD/NAFLD

Metabolic dysfunction-associated steatotic liver disease (MASLD), formerly known as non-alcoholic fatty liver disease (NAFLD), is an updated term to describe fatty liver disease linked to metabolic syndrome. It represents the most prevalent form of chronic liver disease and is recognised as the most prominent contributor to liver-related morbidity and mortality [97]. Data show that the worldwide pooled prevalence of MASLD in patients with IBD is 30.7%, and there is no significant difference in the risk of developing MASLD between ulcerative colitis and Crohn’s disease patients [98].

MASLD is characterised by the abnormal accumulation of fat within liver cells in the absence of significant alcohol consumption. It is typically seen in patients with metabolic risk factors, such as type 2 diabetes (T2DM), obesity, hypertension, and dyslipidaemia [99]. It has an asymptomatic presentation; however, patients show non-specific symptoms such as abdominal discomfort, fatigue, and anxiety [100].

### The influence of IBD on MASLD/NAFLD development and progression

Inflammation and gut dysbiosis in IBD result in increased intestinal permeability, enabling microbial products, such as lipopolysaccharides and toxins, to translocate to the liver via the portal vein. This results in the release of pro-inflammatory cytokines in the liver, causing oxidative stress and hepatic injury. This chronic low-grade hepatic inflammation contributes to MASLD pathology [101, 102]. This is compounded by the fact that many of the risk factors associated with MASLD, such as obesity, are associated with systemic inflammation, which further aggravates the liver disease [103].

IBD is a recognised risk factor for MASLD development, even in lean individuals. A cross-sectional, case-control study measured MASLD prevalence in lean IBD patients compared to lean non-IBD controls. The study found MASLD prevalence to be significantly higher in the IBD group than in the control group (21.3% and 10%, *p* = 0.022), concluding that IBD is an independent risk factor for MASLD in lean individuals, even after accounting for factors like traditional metabolic risk factors (such as obesity) and prior steroid use [104]. However, this study was limited by its dependence on non-invasive diagnostics for MASLD [104]. Nonetheless, this finding of a higher MASLD prevalence in IBD patients aligns with other studies [105–108].

While IBD increases the risk of MASLD development, it is unclear whether it affects the severity or progression of established MASLD. A retrospective cohort study examined 84 patients with co-existing IBD-MASLD. It concluded that there was no association between IBD extent or severity, and MASLD severity. However, this study was limited by the absence of a MASLD-only control population [109]. In contrast, a retrospective study that examined 78 patients with coexisting IBD-MASLD (compared to 145 MASLD-only patients) found that IBD severity correlated with MASLD severity, with greater IBD severity correlating with greater MASLD severity [110].

These findings were contradicted by another study that compared 65 underweight and 65 normal-weight IBD patients, using weight as a marker for IBD activity. They found that underweight IBD patients likely had higher disease activity and predominantly had mild to moderate steatosis rather than severe steatosis [111]. Therefore, they concluded that IBD severity may not necessarily correlate with MASLD severity. However, a key weakness of this study was that they were unable to directly assess the relationship between IBD severity and MASLD severity, as they relied on weight as an indirect marker of IBD severity. As of now, it is unclear whether weight is a reliable marker of IBD severity [112–116].

In contrast, a prospective study found that lean IBD patients had more severe hepatic steatosis, higher levels of systemic inflammation, increased intestinal permeability, and subsequent hepatic inflammation, suggesting an inflammation-driven gut–liver axis mechanism [108]. Likewise, a study identified IBD and other immune-mediated diseases as independent risk factors for advanced MASLD [117]. A study further reported that ulcerative colitis is associated with accelerated hepatic steatosis progression [118].

Thus, there exists an association between MASLD and active IBD (IBD that is not in remission) [102]. However, the nature of this association is debated. There are mixed findings from retrospective studies, while prospective studies suggest that IBD severity may influence MASLD progression and that IBD severity is associated with MASLD severity. Further high-quality, prospective studies that compare IBD-MASLD patients to MASLD-only controls are warranted to validate this relationship.

### Influence of MASLD on IBD development and severity

Although MASLD is frequently observed as a comorbidity in patients with IBD, recent evidence suggests a potential bidirectional relationship. A large cohort study demonstrated that MASLD significantly increases the risk of developing IBD, with a 2.245-fold higher hazard ratio for diagnosis and a progressive increase in cumulative risk over time [119]. This shows that MASLD may actively contribute to the development of IBD and not just co-exist with IBD.

There is contrasting evidence on whether MASLD affects disease severity and activity in patients with established IBD. While some studies suggest no association between MASLD and IBD disease extent (the proportion of bowel affected) [120], other studies indicate otherwise, suggesting a link between MASLD and increased IBD disease complexity [104]. Liver dysfunction and chronic hepatic inflammation, as seen in MASLD, can disrupt bile acid metabolism and the gut microbiota, collectively leading to intestinal immune dysregulation, including dysbiosis and a defective gut barrier. Therefore, it is plausible that MASLD could exacerbate established IBD or contribute to IBD development [121]. Nonetheless, further studies are needed to clarify the influence of MASLD on established IBD.

### Therapeutic considerations in MASLD patients with IBD

#### The influence of IBD treatment on MASLD

A meta-analysis found no evidence that IBD medications, including immunomodulators such as azathioprine and methotrexate and biologics such as TNF-α inhibitors, have an association with the risk of MASLD. Although this was extended to corticosteroids, the meta-analysis recognised that high-dose or extended corticosteroid therapy may lead to an increased risk of MASLD [29]. It is accepted that glucocorticoids can induce insulin resistance. This resistance impairs glucose uptake and encourages further lipolysis, increasing the amount of free fatty acids in the liver. Glucocorticoids promote lipolysis in the adipose tissues, increasing the release of free fatty acids into the circulation. The fatty acids are then taken up by the liver and are re-esterified into triglycerides, resulting in hepatic steatosis [122].

#### The influence of MASLD treatment on IBD

Currently, the main treatment options for MASLD include lifestyle interventions, such as weight loss and addressing cardiometabolic risk factors [123]. However, lifestyle interventions can be limited by the patient’s adherence to those approaches, likely due to comorbidities. Therefore, there has been increasing attention on the development of pharmacological treatments for MASLD [124].

Currently, various drug classes are under investigation for MASLD treatment, including GLP-1 receptor agonists (GLP1RAs) and FXR agonists [125]. Although no pharmacologic treatment is yet approved for MASLD without steatohepatitis [124], two drugs have been approved for treating MASH (Metabolic dysfunction-associated steatohepatitis) with moderate to advanced liver fibrosis. Specifically, in March 2024, resmetirom, a thyroid hormone receptor-β agonist [126], was the first drug approved by the United States Food and Drug Administration (FDA) [125]. In patients with MASLD/MASH, GLP1RAs significantly improved serum ALT, AST, and GGT. Histologically, GLP1RAs significantly improved lobular inflammation, hepatic steatosis, and hepatocellular ballooning. Moreover, liver stiffness was significantly improved. However, whether GLP1RAs can reverse fibrosis or prevent progression to cirrhosis remains to be determined by future RCTs [127]. Accordingly, in August 2025, the GLP1RA semaglutide was the second drug to be approved by the FDA for the treatment of MASH with moderate-advanced liver fibrosis [128].

To the best of our knowledge, there are presently no studies that have explored the effects of resmetirom or thyroid hormone receptor-β agonists on IBD.

GLP1RAs are not associated with new-onset IBD. These could potentially be protective for new-onset IBD. For established IBD, GLP1RAs were found to be generally safe in IBD patients and may improve inflammation. Many studies suggest that GLP1RAs are associated with no change in IBD activity. For example, GLP1RAs were associated with (a) no increased risk of IBD flares, (b) no change in corticosteroid requirements and (c) no change in advanced therapeutic (including biologics) requirements in IBD patients. These studies collectively indicate no variance in IBD activity [129].

On the other hand, other studies suggest that GLP1RAs are associated with improved IBD activity; for example, GLP1RAs were associated with a decrease in C-reactive protein, a key marker of inflammation, and GLP1RAs were also associated with a decrease in corticosteroid requirements and a decrease in advanced therapeutic requirements. Notably, GLP1RAs were also associated with favourable IBD outcomes. For example, they were associated with reduced hospitalisations, reduced rates of surgeries, and reduced rates of IBD-related complications such as perianal fistulae and colorectal carcinoma. There is some evidence to suggest that GLP1RAs could be associated with worsened IBD activity. GLP1RAs have been associated with an increased need for advanced therapeutics [129], suggesting that worsened IBD activity may have necessitated an escalation in the treatment plan. Importantly, GLP1RAs have gastrointestinal side effects, including but not limited to nausea, vomiting, and diarrhoea. However, these are usually mild, and GLP1RAs can still be safely used in IBD patients [130].

GLP1RAs are associated with improved IBD activity, and the favourable outcomes can be explained by their immunomodulatory actions. For example, GLP1RAs can reduce inflammation through various mechanisms, including the inhibition of NF-κB pathway [131], which is implicated in IBD immunopathogenesis [132]. This results in the reduced production of pro-inflammatory cytokines, including IL-1β, IL-6, and TNF-α [131]. Furthermore, GLP1RAs can also directly bind to GLP-1 receptors on peripheral immune cells, leading to a reduction of C-reactive protein in the blood [133].

Moreover, liraglutide (a GLP1RA) has been found to decrease pro-inflammatory microbial species in T2DM patients, thereby indicating that these can alter the gut microbiota composition. However, clinical studies have been unclear about the GLP1RAs-induced alteration in the gut microbiota composition; for example, the data showed no statistically significant differences between liraglutide and metformin treatments [130]. Nevertheless, as mentioned above, many clinical studies have reported GLP1RAs’ association with no changes in IBD activity, and a small minority of studies suggest an association with worsened IBD activity [129].

Reasons for these discrepancies could be due to the methods used in these studies, including (a) differences in the sample size [130], (b) IBD flare definitions, and (c) dose and duration of GLP1RA usage across studies [129]. Overall, the effects of GLP1RAs on IBD are not fully clear and further clarification is required. Given that many of the studies to date have been retrospective cohort studies, further prospective studies and clinical trials are needed to clarify the effects of GLP1RAs on IBD. Indeed, there are currently two clinical trials investigating the efficacy and safety of GLP1RAs in the Crohn’s disease population [134] and in the IBD-T2DM population [135]. Additionally, there is an ongoing clinical trial investigating the role of semaglutide on intestinal permeability in T2DM patients [136].

In IBD-T2DM patients, there is some evidence that metformin (used for diabetes management) may have a beneficial effect on IBD. A retrospective cohort study found that metformin taken for at least six months improved IBD in patients with T2DM [137]. It could be argued that these findings may be different for MASLD patients, however T2DM is significantly associated with MASLD [138, 139], especially since they both have common pathophysiology [139]. Also, metformin administration in a mouse model of IBD alleviated the IBD [140], but these effects may be short-term, since metformin was only administered for sixteen days. This limits the applicability of the findings to the effects of intermediate and long-term metformin usage on IBD.

Currently, there is no specific treatment strategy for patients with co-existing IBD-MASLD. As the prevalence of IBD as well as MASLD is rising in the younger population [141], it is important that we adopt integrated management strategies and early recognition methods. This will lead to a reduction in morbidity rate and improve long-term outcomes in this subgroup.

Importantly, a co-existing IBD-MASLD is associated with high healthcare utilisation in these patients, including increased hospital readmission rates, longer hospital stays, and higher healthcare costs [142].

## Alcohol consumption, alcohol-related liver disease and IBD

### Overview of alcohol-related liver disease

Alcohol-related liver disease (ARLD) refers to liver damage caused by excessive alcohol consumption, and it encompasses the stages of hepatic steatosis, alcoholic hepatitis, and cirrhosis. Early stages of the disease may be reversed with prompt alcohol abstinence. However, continued drinking can lead to progressive liver injury and life-threatening complications, such as cirrhosis [143]. The prevalence of alcohol-related liver disease in the general population is estimated to be 3.5% [144].

A Swiss IBD cohort study reported that 41.3% of IBD patients actively consumed alcohol, with subgroups such as male smokers over the age of 50 being closely associated with excessive alcohol consumption [145]. However, full statistics regarding ARLD in the IBD population are not known. This is partly because of the challenges in ARLD diagnosis, which include the fact that there is no definitive diagnostic test. Moreover, patients are not always transparent about their alcohol consumption. Also, clinical symptoms of ARLD are usually prominent in later stages of the disease course [146–148].

### How do alcohol consumption and alcohol-related liver disease affect IBD?

For new-onset IBD, alcohol has been identified as a potential risk factor, and no safe level of consumption has been determined [149]. For established IBD, alcohol can worsen IBD. Patients with inactive IBD reported that alcohol (amount consumed similar to the general United States population) worsened their gastrointestinal symptoms. However, the severity of gastrointestinal symptoms did not correlate with how much alcohol was consumed [150]. Excessive alcohol intake has been associated with the development of inflammation and worse IBD outcomes. Interestingly, some alcoholic beverages, such as red wine, when taken in moderation, may improve inflammation [151]. Overall, reducing alcohol intake, especially among those who are currently drinking in excess, may be beneficial for improving IBD. This action can also ameliorate ARLD, given that alcohol cessation is the primary treatment choice [152].

Alcohol-associated bowel disease (ABD) refers to the damaging effects caused by alcohol consumption on the small and large intestines. Thus, ABD could explain why alcohol may worsen IBD pathogenesis. The pathological changes of ABD include (a) intestinal immune dysfunction, (b) increased intestinal permeability, and (c) dysbiosis, as elaborated below [153].

#### Intestinal immune dysfunction and increased intestinal permeability

There is emerging evidence that excessive alcohol consumption is associated with intestinal immune dysfunction that may lead to increased intestinal permeability, followed by microbial translocation to the liver. For example, the CD8^+^ T resident memory cells play a vital role in intestinal immunosurveillance by defending against microbial attachment, invasion, and translocation. In a study, relative to controls and NAFLD patients, ARLD patients showed reduced CD8^+^ T resident memory cells (driven by apoptosis) in the duodenum, and exhibited elevated levels of sCD14 (soluble CD14, a surrogate marker of microbial translocation), where these levels inversely correlated with CD8^+^ T cell numbers [154]. Thereby, ARLD could lead to defective microbial immunosurveillance in the duodenum and facilitate microbial translocation.

Another example is Goblet cell-associated antigen passages (GAPs). These exhibit a protective immune response by allowing sampling of luminal antigens by the lamina propria antigen-presenting cells. Chronic alcohol consumption in humans can reduce the formation of GAPs and disrupt normal antimicrobial immune surveillance in the gut. Unsurprisingly, alcohol-fed mice with inhibited GAP formation showed increased bacterial translocation to the liver [155].

Chronic alcohol abuse can cause subclinical intestinal inflammation and TNF-α production by the increased numbers of monocytes and macrophages in the lamina propria of the intestine. The subsequent TNF receptor-1-mediated activation of the myosin light-chain kinase in the intestinal epithelial cells leads to loss of epithelial tight junction proteins [20] contributing to increased intestinal permeability [156]. However, inflammatory cell expansion may not fully account for alcohol-induced intestinal permeability. For instance, the overall number of duodenal CD68^+^ macrophages in AUD (Alcohol Use Disorder) patients was found to be reduced compared with controls [157]. Also, an alcohol binge can activate the cannabinoid receptor-1 on intestinal epithelial cells and ERK1/2 signalling, leading to downregulation of tight junction proteins, which may increase intestinal permeability [158].

Collectively, these findings suggest that alcohol, in the context of ARLD, can lead to compromised intestinal immune surveillance and impaired pro-inflammatory intestinal activation status [155, 159]. This may result in alcohol-induced microbial translocation [154, 155] which could worsen the inflammation in IBD, given that microbial translocation plays a major role in IBD pathogenesis [160, 161].

Notably, alcohol-induced paracellular intestinal permeability may not be the primary driver of microbial translocation. A study in AUD patients found that surrogate markers of microbial translocation were similarly raised in those with increased and normal gut permeability. Instead, intestinal immune dysfunction, including impaired immune surveillance, appears to be more relevant for the gut barrier dysfunction and subsequent microbial translocation [162].

#### Dysbiosis

Alcohol consumption can also result in gut dysbiosis. It was found that alcohol promoted an increase in pro-inflammatory bacteria and simultaneously decreased anti-inflammatory bacteria in the gut [163]. Notably, *Clostridioides difficile*, a pro-inflammatory bacterium [164], can colonise and proliferate in the context of alcohol-induced dysbiosis [151]. Indeed, *C. difficile* can harm intestinal epithelial cells and induce inflammation via the proinflammatory cytokines [165]. It was found that alcohol abuse disorder patients harboured a microbial signature similar to that of IBD patients [166]. Taken together, these findings suggest that alcohol-induced gut dysbiosis may facilitate IBD pathogenesis.

#### Convergent intestinal pathologies can exacerbate disease: IBD-ARLD

Following consumption, generally, higher levels of alcohol are found in the duodenum and jejunum, compared with other distal intestinal segments such as the ileum, cecum, and colon [153]. This could explain why AUD patients showed increased intestinal permeability, primarily in the duodenum and jejunum, with limited changes in the ileum and colon [162]. As such, alcohol induced intestinal immune dysfunction and increased intestinal permeability have chiefly been characterised in the duodenum [154, 155, 162], although alcohol can disrupt barrier function along multiple regions of the gut.

Importantly, ulcerative colitis typically involves continuous inflammation of the colon, which usually starts at the rectum [5], while Crohn’s disease is characterised by discontinuous skip lesions that can affect any segment of the gut, though the distal ileum is mostly affected [6]. Therefore, in co-existing IBD-ARLD, alcohol-induced proximal bowel pathology may occur in parallel with IBD-induced inflammatory lesions at other intestinal sites. Both proximal and distal gut segments could be concurrently affected, synergistically increasing microbial translocation and exacerbating liver injury; the situation is further compounded by alcohol-induced dysbiosis. Nevertheless, the spatial relationship between these processes remains to be determined, including whether alcohol-associated bowel pathology overlaps with active Crohn’s disease within the same intestinal segment.

### Effect of IBD on ARLD

To the best of our knowledge, there are no studies that have directly explored the effects of IBD on ARLD. Further research is required in this area to explore the impact of IBD on ARLD, which could lead to specific treatment strategies to manage coexisting IBD-ARLD. However, we know that alcohol-induced intestinal immune dysfunction, increased intestinal permeability, and gut dysbiosis result in the uncontrolled translocation of microbial products to the liver via the portal vein, resulting in pathogenic immune responses and hepatic inflammation [20, 167–169], which can contribute to ARLD pathogenesis [20].

### Therapeutic considerations of ARLD/chronic and/or excessive alcohol consumption in IBD patients

Although IBD medications and alcohol are risk factors for hepatotoxicity [170, 171], there are no specific guidelines for managing IBD-ARLD patients. Therefore, management based on the best practices for each respective condition is currently employed. Reducing alcohol intake would address the ARLD, limit the interactions between alcohol and IBD medications [151], and reduce the effects of alcohol in IBD patients who drink excessively.

### Summary and clinical implications of ARLD in IBD

Patients who consume excessive alcohol may experience accelerated progression of the liver disease as well as restricted therapeutic options. The hepatotoxic nature of alcohol can determine the use of immunosuppressants, such as methotrexate or biologics, which have known hepatic side effects. As a result, healthcare providers may be forced to modify or withhold effective treatments, potentially resulting in suboptimal control of IBD. This emphasises the importance of screening for alcohol use in IBD patients and modifying treatment plans to minimise hepatic risk [166].

## Gallstone disease and IBD

### Overview of gallstone disease

Gallstone disease is a common digestive condition. It is characterised by the formation of stones in the gallbladder or biliary ducts due to an imbalance in the composition of bile, leading to precipitation of bile components, such as cholesterol and bilirubin [172]. Most gallstones are asymptomatic. Symptoms, when present, manifest as sudden right upper quadrant and/or epigastric pain, which may radiate to the upper back. It frequently results in high healthcare expenditure [173, 174].

### How does IBD affect gallstone disease?

Crohn’s disease has a well-established link to gallstones, affecting about 14.7% of Crohn’s disease patients. Risk factors for gallstone disease include age, Crohn’s disease duration, and lifetime surgery [28]. Furthermore, a meta-analysis found that ulcerative colitis is also significantly associated with a higher gallstone prevalence when compared to controls [175].

IBD is a risk factor for gallstone development. Terminal ileal inflammation in IBD leads to decreased bile acid reabsorption by the enterohepatic circulation. Unabsorbed bile acids in the intestine increase the risk of developing cholesterol gallstones and pigment gallstones in the gallbladder. The risk of gallstones is also increased due to dysbiosis seen in IBD or reduced gallbladder motility associated with Crohn’s disease. This leads to bile stasis, increased crystallisation and stone formation alongside abnormal bile acid metabolism [176, 177].

### How does gallstone disease affect IBD?

Gallstone disease has recently been identified as a risk factor for IBD development. A meta-analysis examined three prospective cohort studies from the UK and the US. The authors found that gallstone disease increased the risk of developing IBD, Crohn’s disease, and ulcerative colitis by 38%, 68%, and 24%, respectively. Like in the case of IBD, gallstones can also lead to abnormal bile acid metabolism, inflammation, and gut microbiota disturbances. Essentially, gallstones disrupt bile acid metabolism by interfering with bile secretion and its bioactive components, which could lead to intestinal inflammation. Therefore, gallstone disease may be a risk factor for IBD [178]. However, further studies are needed to elucidate the underlying mechanisms.

### Therapeutic considerations of gallstones in IBD patients

#### The effect of IBD treatment on gallstones

There is some preliminary evidence that IBD medications may increase the risk of gallstone disease. A meta-analysis found that three or more courses of corticosteroid treatment (odds ratio 2.65) and immunomodulators (odds ratio 1.94) were significantly associated with an increased risk for gallstone disease in Crohn’s disease patients. However, this meta-analysis only included two studies to calculate the odds ratio for each drug class. Moreover, a fixed-effects model was utilised here, which limited generalisability, especially since the ulcerative colitis population was not investigated [28]. In addition to corticosteroids and immunomodulators, the immunosuppressant azathioprine may also increase the risk of gallstone disease. A case report described that azathioprine resulted in cholestatic hepatitis [179]. Given that impaired bile flow can increase the risk of gallstone formation [180], this has implications for all IBD patients because azathioprine is a commonly used IBD drug [181]. Notably, in that case report [179], the patient did not have IBD, limiting its wider applicability to all IBD patients. Furthermore, it was recognised that cholestatic hepatitis is a rare complication of azathioprine use [179]. Whether IBD medications certainly increase the risk of gallstone disease needs to be clarified for both Crohn’s disease and ulcerative colitis populations.

#### The effect of cholecystectomy on IBD

Cholecystectomy (a surgical procedure to remove the gall bladder) can have opposite effects on IBD, depending on the IBD subtype. A Mendelian randomisation study found that cholecystectomy may causally reduce the risk of ulcerative colitis [182]. This was attributed to secondary bile acid accumulation from cholecystectomy, which can relieve colonic inflammation by inhibiting the recruitment of monocytes and macrophages in mouse models [182, 183]. On the other hand, a clinical prospective study found that cholecystectomy was associated with higher disease activity in Crohn’s disease patients [184]. Together, these findings suggest that cholecystectomy may hinder ulcerative colitis development but worsen the Crohn’s disease course.

One reason for this ulcerative colitis- and Crohn’s disease-related discrepancy could be due to the pathophysiological differences between the two diseases. It was shown that secondary bile acids can ameliorate colonic inflammation in mouse models of colitis [185] and can therefore improve ulcerative colitis. However, in Crohn’s disease, the ileum is typically involved [186], which is normally the main site of bile acid reabsorption [187]. This means that in active Crohn’s disease, there is impaired reabsorption of bile acids from the ileum, and so excessive levels of bile acids reach the large intestine. Here, bile acids act as a stimulant, increasing water and electrolyte secretion into the intestinal lumen, and resulting in symptoms such as abdominal pain and watery diarrhoea, known as bile acid malabsorption [188]. Cholecystectomy can also result in excessive levels of bile acids in the intestine, as there is no longer a storage site for bile. Relaxation of the sphincter of Oddi, secondary to gallbladder removal, means there is a continuous release of bile to the gut and subsequently increased generation of secondary bile acids in the large intestine [184].

Excessive bile levels reaching the large bowel can be treated using anion exchange resins, such as cholestyramine and colesevelam, which bind to bile acids with high affinity, increasing the excretion of bile acids into the stool and thereby preventing colonic irritation [189].

#### Cholecystoduodenal fistula and IBD

An important, but rare complication of chronic gallstone disease is a cholecystoduodenal fistula. A cholecystoduodenal fistula is an abnormal and direct anatomical connection between the gallbladder and the duodenum [190–193]. Fistula formation can occur here when chronic gallstone disease induces inflammation, which can lead to ischaemia and ultimately necrosis of the gallbladder wall [190]. The inflamed gallbladder subsequently adheres to the adjacent duodenum, where a gallstone can erode through the wall to form a cholecystoduodenal fistula [190]. The established fistula can allow passage of gallstones to the duodenum, leading to potential gallstone ileus (gallstone mechanically obstructing the intestine) [194].

A cholecystoduodenal fistula has important implications in the Crohn’s disease population. One of the primary sites of gallstone ileus (blockage) in the small intestine is the terminal ileum, owing to its narrow lumen [195]. This is important because the ileum is also most commonly affected in Crohn’s disease [196]. In gallstone ileus, the surgical treatment involves the removal of the gallstone and searching for other gallstones or abnormal fistulae. In Crohn’s disease, given that the gallstone is impacted in the diseased bowel (which may also be strictured), the surgical treatment has a greater focus on the bowels, for example, resecting the bowel or widening it. Moreover, cholecystectomy and closure of the cholecystoduodenal fistula are not recommended in an emergency setting in Crohn’s disease patients [194]. Given that gallstone ileus is considered a surgical emergency that has a high mortality when left untreated [193], further research is necessary to explore optimal management strategies in IBD patients with gallstone ileus.

A potential bidirectional relationship between Crohn’s and gallstone ileus (secondary to cholecystoduodenal fistula) can be postulated. The sustained, transmural inflammatory nature of Crohn’s disease increases the likelihood of entero-enteric, entero-cutaneous or entero-vesical fistulae. It is estimated that 35–40% of Crohn’s disease patients will acquire a minimum of one fistula throughout their disease course [197]. On this basis, we can hypothesise that there could be a greater chance for cholecystoduodenal fistulae formation and subsequent gallstone ileus in Crohn’s disease patients. This hypothesis is supported by the prevalence of gallstone disease observed in the Crohn’s disease population [175] alongside the tendency to fistulate in Crohn’s disease [197]. Nevertheless, further studies are needed to confirm this hypothesis.

## PSC and IBD

### Overview of PSC

Primary Sclerosing Cholangitis (PSC) is a chronic, progressive cholestatic disease that causes fibrosis and inflammation of the intrahepatic and extrahepatic bile ducts, likely due to an abnormal immune response in genetically susceptible individuals. The prevalence of PSC in IBD patients is 2.16% (UC 2.47%, CD 0.96%) [198]. This co-existence of these diseases, recognised as a clinically distinct phenotype IBD-PSC, is more commonly observed in males and the North American and European regions [199]. In the early stages of the disease, it can be asymptomatic, except for an isolated rise in liver function tests (rise in aspartate aminotransferase and alanine aminotransferase by 2–3 times above the upper limit, and rise in alkaline phosphatase (ALP) by 3–10 times above the upper limit) [200]. As the disease progresses, patients may present with abdominal pain, jaundice, pruritus, and fatigue [201]. Over time, the disease can lead to cirrhosis, liver failure, and cholangiocarcinoma [202]. Cholangiography displays the classic beads-on-a-string appearance of the bile duct, caused by alternating segments of bile duct dilation and fibrosis [202].

### How does IBD affect PSC development and progression?

Some studies suggest that IBD may have a protective effect on PSC. For example, a study by Stumme et al. found that liver stiffness values (measured by FibroScan) were significantly lower in patients with co-existing IBD-PSC when compared to PSC-only patients [203], suggesting that IBD may attenuate liver fibrosis and PSC progression. This is corroborated by a retrospective study by Rennebaum et al., where PSC in 41 PSC-only patients showed accelerated disease progression compared to 115 IBD-PSC patients, and PSC-only patients had a higher risk of developing cirrhosis [204].

In contrast, a retrospective study by Liu et al. found no differences in the rates of liver transplantation between IBD-PSC and PSC-only patients. Also, there were no differences in how long patients survived without a liver transplant (liver transplant-free survival rates), between IBD-PSC and PSC-only patients [205]. Given that a liver transplant is an indicator of PSC disease severity [206], these findings imply that IBD does not have a protective effect against PSC.

The discrepancy between these studies could be due to the differences in IBD subtypes, as the differences between Crohn’s disease and ulcerative colitis were not considered. Liu et al. analysed IBD as one entity with no subgroup analysis on Crohn’s disease or ulcerative colitis [205], which may have influenced the results. This explanation is supported by a retrospective study by Fevery et al. They found that in large duct PSC, Crohn’s disease-PSC patients lived longer without liver transplant than ulcerative colitis-PSC [207]. This suggests that those with coexisting Crohn’s disease-PSC have better outcomes than those with coexisting ulcerative colitis-PSC. This infers that Crohn’s disease confers higher protection than ulcerative colitis against PSC disease progression. So, Liu et al. may not have observed a difference in the rates of liver transplantation or liver transplant-free survival rates, between IBD-PSC and PSC-only patients because they did not distinguish between the proportion of Crohn’s disease-PSC and ulcerative colitis-PSC patients, which remains unknown in this study [205]. Furthermore, a prospective cohort analysis of 70 non-colectomised PSC patients with histologic intestinal inflammation reported that intestinal inflammation was associated with a reduced risk of liver transplantation or death [208]. This supports the hypothesis that IBD attenuates PSC disease progression. This study elucidated that lipopolysaccharide may induce hepatocellular NF-κB activation, resulting in the suppression of bile acid synthesis and consequently lessening cholestatic liver damage in IBD-PSC mice [208]. Therefore, lipopolysaccharide translocated to the liver (as in IBD) [73] could ameliorate PSC in IBD-PSC. Evidently, this reinforces the gut-liver axis as the biological basis for the effects of IBD on PSC.

Overall, current evidence suggests that IBD, particularly Crohn’s disease, is protective for PSC disease progression. This may have important implications for the management of IBD-PSC patients. This is because effective control of IBD could remove the protective effect of IBD for PSC disease progression, thereby inadvertently accelerating PSC disease progression. However, this must be considered alongside the elevated risks of cholangiocarcinoma and colorectal carcinoma, which are more prevalent in the IBD-PSC population compared to the PSC-only population [204, 205].

### How does PSC affect IBD?

It is known that IBD-PSC is a distinct clinical phenotype where the IBD presents with more extensive disease (often pancolitis). However, paradoxically, IBD-PSC patients exhibit fewer gastrointestinal symptoms, with many experiencing quiescent or asymptomatic IBD despite widespread intestinal inflammation [209]. This may be because PSC could alter the intestinal microbiota in a way that promotes Foxp3^+^ Treg cell expansion in the colon, protecting against IBD [210]. Therefore, this suggests that PSC attenuates IBD.

The clinical disease course in the IBD-PSC population is often mild, with ulcerative colitis-PSC patients requiring fewer steroids and colectomies than those with ulcerative colitis alone [211]. However, although the IBD itself is typically milder in IBD-PSC than in those with IBD alone [212], IBD-PSC is associated with a significantly increased risk of dysplasia and colorectal cancer compared to those with IBD alone [213]. The phenomenon may be driven by a unique immune response characterised by the expansion of specific CD4^+^ T cells and IgG-secreting plasma cells, which create a pathogenic environment in the colon that is associated with a greater risk and faster progression to dysplasia and cancer. Therefore, patients with IBD-PSC require specialist follow-up and surveillance for colorectal cancer [214].

### Therapeutic considerations in IBD-PSC

Despite the immune-mediated nature of PSC, current evidence suggests that immunosuppressants (used in IBD) may not offer any clinical benefit or improve survival in PSC [215]. However, glucocorticoids have been shown to improve ALP levels in PSC [216]. ALP is a prognostic marker of PSC, with lower ALP levels associated with slower disease progression and fewer complications [217, 218]. If an immunosuppressant agent like a glucocorticoid can lower ALP levels in PSC, then it is conceivable that this might improve PSC outcomes. However, existing evidence is scarce and equivocal, so further research is required to clarify this important clinical question.

Certain biologics used in IBD appear not to affect PSC. A retrospective analysis explored the efficacy of the anti-TNF biologics infliximab and adalimumab in 141 PSC patients over 3 to 12 months. They measured the response of patients’ IBD (endoscopic appearance or symptom profile) to the drugs and examined serum ALP [219], an important PSC biomarker [220]. Data showed that while 48% of patients demonstrated a response to these anti-TNF therapies for their IBD, there were only short-term changes with ALP. Specifically, when compared to baseline ALP levels, adalimumab displayed significant reductions in ALP at 3 and 6 months; however, both infliximab and adalimumab displayed no significant ALP changes at 12 months [219]. The overall lack of ALP alterations in the long-term with IBD biologics may be due to the limited study size. However, a subsequent meta-analysis corroborated the study by concluding that infliximab and adalimumab do not improve cholestasis biomarkers, including ALP [221]. In summary, existing evidence suggests that biologics used to treat IBD may not affect ALP levels in PSC.

Note that the co-existence of IBD and PSC is a distinct phenotype that differs from IBD alone [212] and that IBD within IBD-PSC is usually less severe than IBD alone, which could explain why IBD-PSC patients have a good response to mesalamine (a first-line drug for ulcerative colitis) for their IBD, when IBD is not too advanced [212]. Accordingly, a minority of IBD-PSC patients require advanced therapeutics, such as biologics [212]. However, the optimal advanced therapeutic choice for co-existing IBD-PSC is not currently established [222]. For example, the response rate to the biologic vedolizumab for IBD in IBD-PSC patients was similar to that of those with IBD alone [223]. However, despite being beneficial for their IBD, it was associated with adverse liver enzyme outcomes (significant rise in ALP). This suggests that vedolizumab could exacerbate PSC disease progression. A limitation of this study was that there was no cross-validation, via histology or imaging, to further support this biochemical finding [221]. Overall, there is limited high-quality evidence that could inform which advanced therapy would be most efficacious to utilise in co-existing IBD-PSC [222].

A randomised controlled trial investigating whether a particular biologic is superior in treating IBD within the IBD-PSC population would greatly aid in clarifying the optimal advanced therapeutic regimen for IBD in the IBD-PSC population. Importantly, this could also clarify whether a particular biologic may improve or worsen the PSC disease course.

The lack of evidence base for pharmacological treatments for co-existing PSC and IBD highlights the vital role of liver transplantation in the IBD-PSC population [221].

### Further therapeutic considerations: liver transplantation in IBD patients

Approximately 40% of PSC patients need a liver transplant during their lifetime [224]. Thus, this section discusses the variability in post-liver-transplantation outcomes in IBD patients and the role of immunosuppressants in IBD outcomes.

IBD outcomes following liver transplantation are variable. An earlier review examined 14 studies with a total of 609 IBD patients who received a liver transplant for PSC, with a median follow-up of approximately 4.8 years. It identified three distinct outcomes: 39% of IBD patients showed no observable effects following liver transplantation, 31% of IBD patients showed improvement of IBD, and 30% showed worsened IBD [225]. A subsequent meta-analysis found no changes in IBD following the liver transplant (51.4%). Amongst the post-liver-transplantation outcomes, IBD improvement was the second most common outcome (29.4%), and IBD worsening was the least common outcome (25.2%) [226]. A more recent meta-analysis found that 17.8% of IBD patients showed worsened IBD following liver transplant. Although this study did not report on the other IBD outcomes, the incidence of IBD worsening after liver transplant seems to be in keeping with the other studies [227].

Although both meta-analyses were limited by considerable heterogeneity between the included studies [226, 227], current evidence suggests that after a liver transplant, no effect on IBD is the most likely outcome, followed by IBD improvement, and lastly, IBD worsening. These findings apply to patients who were already diagnosed with IBD at the time of liver transplantation, since it is known that *de novo* IBD can arise post liver transplantation [225, 227]. Thus, IBD patients should be counselled on the risks and benefits of undertaking liver transplant, importantly, about IBD exacerbation and IBD improvement, respectively.

#### Possible role of immunosuppressants in determining IBD outcomes

The determinants of the three IBD outcomes (no change to IBD, improved IBD, worsened IBD) in patients following their liver transplant are not fully understood. Certain immunosuppressants administered following liver transplant have been implicated in IBD worsening, specifically tacrolimus [228, 229] and tacrolimus-based regimens, such as tacrolimus and mycophenolate mofetil [230]. In contrast, other immunosuppressants have been associated with IBD improvement, notably cyclosporin [231], azathioprine [228], and the combination of cyclosporin A and azathioprine [230]. However, there is an inconsistency between studies; for example, another study found that tacrolimus may not worsen IBD [232]. Further studies are needed to determine optimal immunosuppressant regimens that would limit liver transplant rejection, prevent IBD worsening, and show minimal drug-induced side effects.

Table 1 presents the effects of different immunosuppressants on IBD outcomes following liver transplant.

**Table 1:** The effects of immunosuppressants on IBD following liver transplantation

Immunosuppressant treatment following liver transplant	IBD outcome
**Non biologics**	
**Tacrolimus-based regimen**	**May worsen IBD** Tacrolimus, tacrolimus-based regimens and dual therapy with tacrolimus and mycophenolate mofetil were associated with an increased risk of IBD exacerbation [228–230, 233]. **May not change IBD** • No significant differences were found in the rates of ulcerative colitis relapses or corticosteroid requirements in tacrolimus users when compared to ciclosporin users [234]. • Tacrolimus was not predictive of recurrent IBD. However, there was a non-significant trend towards an increased risk of recurrent IBD in tacrolimus users when compared with cyclosporin users [232].
**Azathioprine-based regimen**	**May improve IBD** • Azathioprine was associated with a reduced risk of IBD exacerbation [228, 233]. • Dual therapy with azathioprine and cyclosporin A was associated with a reduced risk of IBD exacerbation [230].
**Cyclosporin-based regimen**	**May improve IBD** • Cyclosporin was associated with a reduced risk of ulcerative colitis exacerbation [231]. • Dual therapy with cyclosporine and prednisolone was associated with a reduced risk of ulcerative colitis exacerbation [235]. • Dual therapy with azathioprine and cyclosporin A was associated with a reduced risk of IBD exacerbation [230].
**JAK inhibitors**	**May improve IBD** A case report found that tofacitinib resulted in remission of severe ulcerative colitis that was refractory to anti-TNFs, vedolizumab, and Ustekinumab [236].
**Biologics**	
**Anti-TNF therapy**	**May improve IBD** • Infliximab was shown to result in remission of IBD [237] including ulcerative colitis [238, 239] as well as improvement in IBD [240–242] including ulcerative colitis [239, 243]. • Adalimumab demonstrated remission of IBD [240] including in Crohn’s disease that was refractory to tacrolimus [244] and also showed improvement in IBD [240, 241]. • Infliximab with subsequent adalimumab resulted in remission of IBD [240] and improved IBD [241, 245]. • Adalimumab with subsequent infliximab resulted in remission of IBD and improved IBD [240]. • Furthermore, a case series also reported that in a patient on adalimumab, it resulted in improved IBD, but the patient’s erythema nodosum worsened. The patient was then switched to infliximab, which improved the erythema nodosum [237]. **May not change IBD** • Infliximab did not demonstrate a clinical response [240, 246], including in a patient with worsening ulcerative colitis [239]. • Similarly, adalimumab showed no response [240] including in a patient with worsening Crohn’s disease [239]. • Several case studies reported that treatment with infliximab and then adalimumab demonstrated no clinical response [240, 241, 246]. **May worsen IBD** • Initially, infliximab, adalimumab, and adalimumab with subsequent infliximab demonstrated some clinical response. However, by the end of treatment or the most recent follow-up, these drugs resulted in a loss of response in IBD patients [240]. • A case report found a patient with a severe flare of Crohn’s disease one year after commencing certolizumab. The Crohn’s disease was previously refractory to adalimumab and infliximab [247].
**Interleukin antagonists**	**May improve IBD** Case reports found that ustekinumab was safe and effective in inducing remission for (a) Crohn’s disease that was refractory to anti-TNFα treatment, and (b) ulcerative colitis following discontinuation of vedolizumab [247, 248].
**Anti-integrin agents**	**May improve IBD** Several case studies have reported that vedolizumab resulted in improved IBD in three out of five patients [249], demonstrated a clinical response in four out of ten patients, including remission in one patient [250], induced remission for ulcerative colitis that was refractory to azathioprine and infliximab [251], and clinically improved IBD [252] that was refractory to other biologics [245].

## Future research directions

Given the notable comorbidity of IBD and liver disease, further studies are necessary to elucidate this underlying connection. Exploring the direct effects of IBD on ARLD would be useful. Research is needed to determine the effect of IBD on already established MASLD, and the mechanisms underlying the increment of IBD risk by gallstones. With regards to treatment, clarification is needed on the effect of IBD medications on MASLD and PSC, as well as on the optimal immunosuppressive regimen in IBD patients who have undergone liver transplantation. Such studies will help inform the management of coexisting IBD and liver pathology.

## Summary points and summary table

This review addresses the physiological and pathological connections between the liver and gut, specifically in the context of IBD and some common hepatobiliary pathologies.

1. Physiologically, bile acids facilitate the endocrine and immunological links between the gut and liver. For example, GLP-1 secretion from the gut is mediated via bile acid-induced TGR5 activation on the intestinal L cells. Moreover, primary and secondary bile acids regulate gut microbiota and host immune state via FXR signalling in the intestinal lumen. FXR, expressed in both hepatocytes and enterocytes, regulates bile acid synthesis. In the liver, this involves the inhibition of CYP7A1 and CYP8B1. In the intestine, FXR regulates bile acid transport and influences the intestinal epithelial barrier integrity. Through these coordinated signalling mechanisms, bile acids act as metabolic integrators, allowing the liver to exert broad regulatory effects on intestinal and systemic physiology under normal/homeostatic conditions.
2. Gut dysfunction can damage a healthy liver. For example, chronic inflammation in IBD compromises the intestinal barrier integrity. This permits the translocation of microbial products and immune-derived signals to the liver, contributing to hepatic inflammation. Conversely, a liver pathology can adversely affect a healthy gut. Liver disease can reduce bile acid excretion into the gut, resulting in an altered gut microbiota composition with subsequent disruption of gut homeostasis. Disrupted bile acid production, flow, and metabolism processes compromise the amount of bile acids reaching the intestine, leading to impaired fat digestion and fat absorption from the gastrointestinal tract. Alongside this, decreased FXR and TGR5 activation can compromise the intestinal barrier. Importantly, gut dysbiosis and an impaired intestinal barrier can lead to further hepatic inflammation in a self-perpetuating cycle.
3. PSC and other liver pathologies may necessitate liver transplantation, which usually does not influence IBD, can sometimes improve it, and rarely worsens it. Similarly, immunosuppressive regimens following liver transplantation can have variable outcomes on IBD.

These bidirectional relationships highlight the role of the gut-liver axis as a fundamental framework that can help us understand the pathophysiological links and guide therapeutic strategies in co-existing IBD-hepatobiliary conditions. Further summary in relation to co-existing gut and hepatobiliary conditions is provided in Table 2.

**Table 2:** Summary of hepatobiliary conditions that can co-exist with IBD

Liver Condition	Prevalence of hepatobiliary conditions in IBD patients	How IBD affects hepatobiliary conditions	How hepatobiliary conditions affect IBD	Important clinical considerations when hepatobiliary conditions and IBD co-exist
**MASLD**	As reported in 2022, the worldwide pooled prevalence of MASLD in patients with IBD was 30.7% [98]. Additionally, there is no significant difference in the risk of developing MASLD between the ulcerative colitis and Crohn’s disease patient populations [98].	Prolonged inflammation and gut dysbiosis seen in IBD can result in increased intestinal permeability, enabling microbial products to translocate to the liver, eventually resulting in a pro-inflammatory environment in the liver, causing hepatic injury and aggravating liver disease, especially in the presence of metabolic risk factors [103]. IBD is a risk factor for MASLD development, including in lean individuals [104]. However, it is currently unclear whether IBD influences the disease course in established MASLD.	MASLD disrupts bile acid metabolism and normal gut microbiota, which could exacerbate established IBD or contribute to IBD development [121]. MASLD is a significant risk factor for IBD development. Specifically, there is a 2.2-fold higher hazard ratio for IBD diagnosis and a progressive increase in the cumulative risk of IBD over time [119]. It is currently unclear whether MASLD influences the course of established IBD.	IBD medications, including immunomodulators and biologics, have not been shown to increase the risk of MASLD. Although high dose or prolonged corticosteroid use may elevate this risk due to induced insulin resistance and increased hepatic fat accumulation [29]. The GLP1RA, semaglutide, has recently been approved by the FDA for MASH with mild-moderate liver fibrosis [128]. GLP1RAs are considered safe to use in IBD patients [130]. Clinical studies have demonstrated that GLP1RAs are associated with no changes in IBD activity, or even improved IBD activity, with limited evidence suggesting GLP1RAs are associated with worsened IBD activity [129]. MASLD is consistently linked to higher healthcare utilisation, such as increased duration and frequency of hospital admissions [142].
**ARLD**	Chronic alcohol consumption may result in ARLD. The prevalence of ARLD in the IBD patient population is not fully clear. Context of alcohol consumption- a Swiss cohort study found that 41.3% of IBD patients actively consumed alcohol. Furthermore, certain subgroups, such as male smokers over the age of 50, were closely associated with excessive alcohol consumption [145].	No studies have directly explored the effects of IBD on ARLD so far. Further research is required in this area. Chronic alcohol consumption in IBD can induce gut dysbiosis and can also result in microbial translocation due to increased intestinal permeability and intestinal immune dysfunction, eventually leading to hepatic inflammation.	Here, we summarise the effect of chronic alcohol consumption on IBD. Chronic alcohol consumption can lead to gut dysbiosis [163], and microbial translocation [158, 162] due to increased intestinal permeability [20, 158], and intestinal immune dysfunction [154, 155, 159], thereby contributing to IBD pathogenesis. Excessive alcohol consumption could exacerbate already established IBD [151]. Alcohol consumption is a potential risk factor for IBD development [149]. While moderate consumption of certain alcoholic beverages, such as red wine, could improve inflammation, alcohol is a potential risk factor for new-onset IBD [149].	Chronic alcohol consumption can accelerate liver disease and limit effective IBD therapeutic options, owing to the hepatic side effects of IBD medications, potentially leading to suboptimal IBD management and limiting IBD therapeutic options. Therefore, screening for alcohol use is important in IBD patients [166].
**Gallstones** (Cholelithiasis)	Crohn’s disease has a well-established link to gallstones, affecting about 14.7% of patients and is driven by factors like age, disease duration and surgery [28]. Ulcerative colitis also shows significantly increased gallstone prevalence versus controls [175].	Inflammation in IBD decreases enterohepatic circulation and reabsorption of bile acids. In turn, unabsorbed bile acids increase the risk of cholesterol and pigment gallstone development in the gallbladder. Other factors that increase the risk of gallstones include reduced gallbladder motility in Crohn’s, which can cause bile stasis and abnormal bile acid metabolism, as well as dysbiosis in IBD [176, 177]. IBD is a risk factor for gallstone disease development [176, 177].	Gallstones increase the risk of IBD development. This is because of abnormal bile acid metabolism, gut microbiota disturbances, and inflammation [178].	IBD disease medications (corticosteroids, immunomodulators and immunosuppressants) may increase the risk of gallstone disease in the Crohn ’s-gallstones population [28]. Cholecystectomy, the surgical treatment for gallstones, may reduce the risk of ulcerative colitis [185], but, in contrast, can worsen Crohn’s disease [184, 188].
**PSC**	The prevalence of PSC in IBD is 2.16% [198]. Co-existence of these diseases, recognised as a clinically distinct phenotype “IBD-PSC”, is more commonly observed in males and the North American and European regions [199].	IBD may attenuate PSC progression, potentially via suppression of bile acid synthesis, mediated by lipopolysaccharide-induced NF-κB activation in the hepatocytes [208]. Some studies show slower fibrosis and less cirrhosis in IBD-PSC patients compared to PSC-only patients, especially in Crohn’s disease-PSC [203, 204]. However, some studies found no difference in disease severity markers such as liver transplant rates, possibly because they did not distinguish between IBD subtypes. Nonetheless, IBD-PSC patients remain at a higher risk of cholangiocarcinoma and colorectal cancer [204, 205]. IBD is protective for PSC disease progression [203, 204].	PSC may attenuate IBD severity by altering the intestinal microbiota via promotion of Foxp3 + Treg expansion in the colon [210]. Thereby, PSC may have a protective effect on IBD.	IBD medications such as immunosuppressants [215] and biologics [221] may not improve the PSC disease course.

## Data Availability

No datasets were generated or analysed during the current study.
